# Synthesis, In Vitro Antioxidant Properties and Distribution of a New Cyanothiophene-Based Phenolic Compound in Olive Oil-In-Water Emulsions

**DOI:** 10.3390/antiox9070623

**Published:** 2020-07-16

**Authors:** Sonia Losada-Barreiro, Matej Sova, Janez Mravljak, Luciano Saso, Carlos Bravo-Díaz

**Affiliations:** 1 Physical Chemistry Department, Chemistry Faculty, University of Vigo, E-36310 Vigo, Spain; cbravo@uvigo.es; 2 REQUIMTE-LAQV, Chemistry and Biochemistry Department, Science Faculty, University of Porto, PT-4169-007 Porto, Portugal; 3 Faculty of Pharmacy, University of Ljubljana, Aškerčeva 7, 1000 Ljubljana, Slovenia; janez.mravljak@ffa.uni-lj.si; 4 Department of Physiology and Pharmacology “Vittorio Erspamer”, Sapienza University of Rome, 00185 Rome, Italy; luciano.saso@uniroma1.it

**Keywords:** cyanothiophene scaffold, oil-in-water emulsion, distribution, interfacial concentration, drug-like properties, antioxidant

## Abstract

We synthesized and determined the antioxidant activity and distribution of a new cyanothiophene-based compound, *N*-(3-cyano-4,5,6,7-tetrahydrobenzo[*b*]thiophen-2-yl)-3,5-dihydroxybenzamide (SIM-53B), in intact stripped olive oil-in-water emulsion. The in vitro antioxidant properties of SIM-53B were evaluated and compared to those for Trolox and resveratrol. Addition of an emulsifier (Tween 20) creates a narrow region, the aqueous–oil interface, and the distribution of SIM-53B can be described by two partition constants: *P*_W_^I^ (between aqueous/interfacial regions) and *P*_O_^I^ (between oil/interfacial regions). The effects of emulsifier concentration expressed in terms of the volume fraction, Φ_I_, and O/W ratio were also evaluated on its distribution. SIM-53B is predominantly distributed (>90%) in the interfacial region of 1:9 (O/W) olive oil-in-water emulsions at the lowest emulsifier volume fraction (Φ_I_ = 0.005) and only a small fraction is located in the aqueous (<5%) and the oil (<5%) regions. Besides, the concentration of SIM-53B in the interfacial region of the emulsions is ~170–190-fold higher than the stoichiometric concentration, emphasizing the compartmentalization effects. Results suggest that the emulsifier volume fraction is a key parameter that may modulate significantly its concentration in the interface. Our study suggests that cyanothiophene-based compounds may be interesting additives for potential lipid protection in biomembranes or other lipid-based systems.

## 1. Introduction

Drug-resistant bacteria emerge as an increasing threat to human health and new antibacterials are needed to treat bacterial infections [1,2]. Bacterial cell wall biosynthesis involves enzymes which have been engaged as targets for developing potential antibacterial candidates [3,4]. In this sense, cyanothiophene scaffold has been used in medicinal chemistry for the preparation of biologically active compounds and for this reason, the isolation and the structural characterization of innovative derivatives, the improvement of new synthetic methods and the evaluation of biological properties are topics of growing interest [2,5,6,7,8]. A broad series of cyanothiophene-based inhibitors of the MurF enzymes along with two crystal structures have been published [2,6] and further 3D-QSAR molecular docking and molecular dynamics studies were also performed [9]. Additionally, 5-((4a*R*,7a*R*)-6-(5-Fluoro-4-methoxy-6-methylpyrimidin-2-yl)-2-imino-3-methyl-4-oxooctahydro-1*H*-pyrrolo[3,4-*d*]pyrimidin-7a-yl)thiophene-2-carbonitrile was found as a potent inhibitor of β-site amyloid precursor protein cleaving enzyme (BACE) [5]. Cyanothiophene-based compounds also showed inhibitory activity on penicillin-binding protein 2a (PBP2a) (unpublished results).

However, even though some of these compounds exhibit potent enzyme inhibitory activity at low concentrations and promising antibacterial activity, the aqueous solubility of these compounds is very low. Thus, their addition to aqueous pharmaceutical formulations may be challenging due to the fact that their low aqueous solubility will lead to poor oral bioavailability. In this regard, new approaches to increase their bioaccessibility and thus bioavailability need to be developed.

Oil-in-water emulsions have gained attention as promising or potential carrier systems to incorporate hydrophobic bioactive compounds such as cyanothiophene inhibitors into aqueous pharmaceutical formulations [10,11,12,13,14,15]. The parenteral method is one of the most frequent and effective routes for hydrophobic drug administration. In recent scientific investigations, nanoemulsions have been frequently employed as a suitable vehicle for the parenteral administration of poorly soluble anticancer agents such as chlorambucil [15], myrisplatin [16], paclitaxel [17], chalcone [18] and didodecyl methotrexate [19] for treatment of different carcinomas. Similarly, carbamazepine nanoemulsions have been developed to enhance its solubility and have been used for CNS targeting of carbamazepine assessing its pharmacokinetic efficiency [20]. Other references reported the use of nanoemulsions for increasing the bioavailability of other compounds such as diazepam, propofol and prostaglandin E1 [21].

**Scheme 1:**
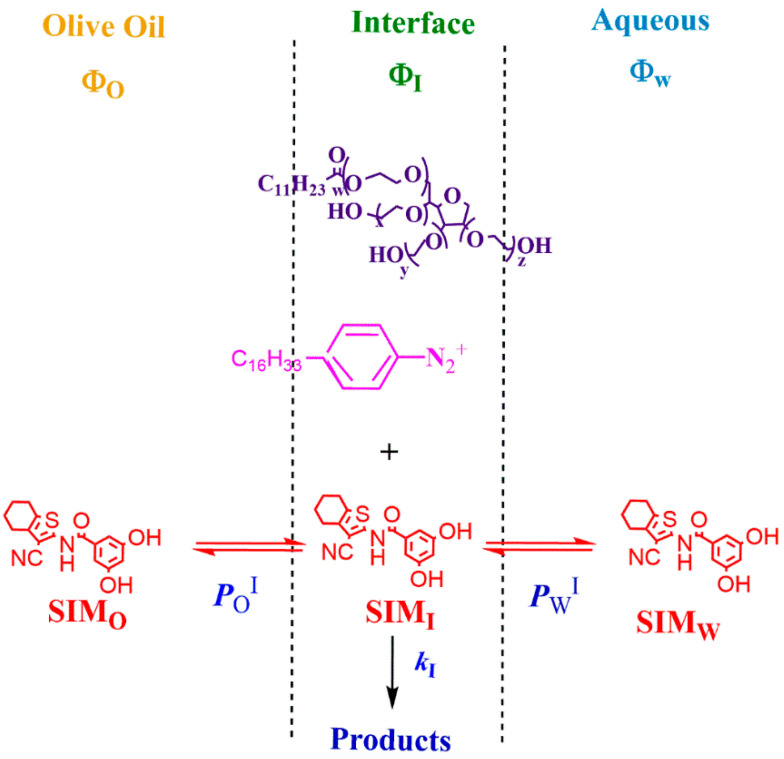
Distribution of SIM-53B between the different regions of the emulsified system, aqueous (W), oil (O) and interfacial (I) regions. Φ is the volume fraction of a particular region (Φ = V_region_/V_total_). *P*_W_^I^ and *P*_O_^I^ are the partition constants between the aqueous and interfacial regions and between the oil and interfacial regions, respectively. *k*_I_ is the rate constant for the reaction between the chemical probe (hexadecylbenzenediazonium ion, 16ArN_2_^+^) and SIM-53B in the interfacial reaction of the O/W emulsion.

Oil-in-water emulsions are opaque mixtures of two immiscible liquids (oil droplets that are dispersed in water) that are thermodynamically unstable that can be stabilized kinetically by emulsifiers such as phospholipids, proteins or Tweens. Conceptually, emulsified systems can be partitioned into three different regions with different solvent characteristics, aqueous (W), interfacial (I) and oil (O) regions, Scheme 1, and loaded compounds can be distributed between the different regions according their hydrophilic–lipophilic balance (HLB) [22]. The interfacial region represents only a small fraction of the total volume of an emulsion and it is well known that interfacial properties are a key factor for determining the physicochemical properties of the emulsion and for the bioavailability of the loaded bioactive compounds [23].

The emulsions can be prepared from a variety of different food-grade lipids including oils (soybean, olive, fish oils), triacylglycerols (TAGs), diacylglycerols (DAGs), monoacylglycerols (MAGs) or mixtures that are biocompatible with biological membranes. However, the oil region may be prone to oxidation due to the presence of oxygen, leading to undesirable and unhealthy radical oxygen species (ROS) [24]. Efforts have been made to enhance the oxidative stability of emulsions by modulating the physical distribution of antioxidants (AOs) into the interfacial region to stop or delay lipid oxidation [22]. In this sense, in our previous work, we employ an innovative methodology to gain insights into the complex structure–reactivity relationships governing the efficiency of AOs in emulsions composed of different oils (soybean, olive, corn and fish) [22]. Several representative series of homologous AOs of different hydrophobicity derived from potent, natural antioxidants (gallic, chlorogenic, caffeic, protocatechuic acids and hydroxytyrosol derivatives) were employed for this purpose [25,26,27,28,29]. For the first time, it was possible to demonstrate that the effectiveness of the series of homologous of AOs is directly related to their distribution (that is, with their concentration in the interfacial region), providing new ideas for the best compression of the phenomenon of inhibition of lipid oxidation in emulsified systems and for the preparation of more effective bioactive compounds.

The present study proposes the utilization of emulsions as a lipid system efficient to improve the bioavailability of a cyanothiophene-based compound such as SIM-53B (*N*-(3-cyano-4,5,6,7-tetrahydrobenzo[*b*]thiophen-2-yl)-3,5-dihydroxybenzamide). The aim of the study was to apply our developed methodology [22] to determine the distribution of SIM-53B in olive oil-in-water emulsions to give a more comprehensive overview of the behavior and functionality of these compounds.

## 2. Materials and Methods 

### 2.1. Chemicals and Reagents

All chemicals and reagents were of the highest purity available and were used as received. They were purchased from Acros Organics, Carlo Erba reagents, Apollo Scientrific, Fluka, Sigma-Aldrich, TCI and Merck. 2,2-Diphenyl-1-picrylhydrazyl (DPPH^•^), neocuproine and polyoxyethylene (20) sorbitan monolaurate (Tween 20) were obtained from Aldrich, Merck and Acros Organics, respectively. 2,2′-Azinobis(3-ethylbenzothiazoline-6-sulfonic acid ammonium salt) (ABTS), Trolox and resveratrol were obtained from TCI. The synthesis of *N*-(3-cyano-4,5,6,7-tetrahydrobenzo[*b*]thiophen-2-yl)-3,5-dihydroxybenzamide (SIM-53B) was monitored using thin-layer chromatography on silica-gel plates (Merck DC Fertigplatten Kieselgel 60 GF_254_) and visualized by UV light and/or stained with the relevant reagents (ninhydrin and FeCl_3_ solution). Flash column chromatography was completed on Merck silica gel 60 (mesh size, 70–230). A Reichert hot-stage microscope was employed to measure melting points. IR spectra were recorded on a Perkin-Elmer FTIR 1600 spectrometer. ^1^H and ^13^C NMR spectra were acquired on a Bruker AVANCE III 400 MHz spectrometer in DMSO-d_6_, with TMS as the internal standard. IR, ^1^H and/or ^13^C NMR spectra of compounds 2, 3 and SIM-53B can be found in the Suplementary material (Figures S1–S8). High-resolution mass spectra were acquired with a Micromass^®^ Q-Tof Premier™ mass spectrometer (Micromass, Waters, Manchester, UK). The purity of SIM-53B was determined by reversed-phase high-performance liquid chromatography (HPLC) analysis on an Agilent 1100 system (Agilent Technologies, Santa Clara, CA, USA) equipped with a quaternary pump and a multiple-wavelength detector G1365 UV-VIS. Agilent Eclipse Plus C18 (5 μm, 4.6 × 150 mm) was used as a column, with a flow rate of 1.0 mL/min, detection at 210, 220, 254 and 280 nm and an eluent system of: A = 0.1% TFA in H_2_O; B = acetonitrile. The following gradient was applied: 0–19 min, 20% B to 90% B in A; 19–20 min, 90% B in A. HPLC chromatogram and area percent report for SIM-53B can be found in the Suplementary material (Figure S9).

Stripped olive oil was used in the preparation of emulsions. Endogenous tocopherols and phenols were removed by washing olive oil (~700 mL) with a 0.5 M NaOH solution (4 × 300 mL), followed by 0.5 HCl solution (2 × 200 mL) and passing it twice through an aluminum oxide column. Total elimination of tocopherols was checked by HPLC according to the IUPAC method 2.432 [27].

Milli-Q grade water (conductivity < 0.1 mScm^−1^) was used for the preparation of all aqueous solutions. The acidity of the aqueous phase of emulsions was controlled by using citric acid/citrate buffer (0.04 M, pH 3.65). Hexadecylbenzenediazonium tetrafluoroborate, 16-ArN_2_BF_4_, was obtained from 4-hexadecylaniline (Aldrich, 97%) under non-aqueous conditions as explained in a published method [22]. *N*-(1-naphthyl) ethylenediamine (NED, Acros Organics) was dissolved in an ethanol/butanol mixture (EtOH/BuOH, 50:50, v/v) to give [NED] = 19 mM.

### 2.2. Synthesis of N-(3-cyano-4,5,6,7-tetrahydrobenzo[b]thiophen-2-yl)-3,5-dihydroxybenzamide (SIM-53B)

**Scheme 2:**
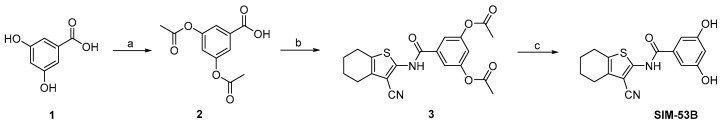
Reagents and conditions. (**a**) Ac_2_O, pyridine, T = 0 °C, then rt 2 h; (**b**) (i) oxalyl chloride, CH_2_Cl_2_, DMF, T = 0 °C, then rt 1 h; (**ii**) CH_2_Cl_2_, 2-amino-4,5,6,7-tetrahydrobenzo[*b*]thiophene-3- carbonitrile, pyridine, T = 0 °C, then rt 24 h; (**c**) MeOH, K_2_CO_3_, rt, 1 h. Abbreviations: rt, room temperature.

The synthesis of SIM-53B started from 3,5-dihydroxybenzoic acid (1) (Scheme 2). The introduction of protecting groups with acetic anhydride afforded 3,5-diacetoxybenzoic acid (2). This was followed by the conversion of an acid 2 to acyl chloride and further addition of 2-amino-4,5,6,7-tetrahydrobenzo[*b*]thiophene-3-carbonitrile, which was prepared by a previously published procedure [2], to obtain 5-((3-cyano-4,5,6,7-tetrahydrobenzo[*b*]thiophen-2-yl) carbamoyl)-1,3-phenylene diacetate (3). The deprotection with potassium carbonate in methanol lead to the final product SIM-53B (Scheme 2).

The detailed synthetic procedures for compounds 2, 3 and SIM-53B (Scheme 2) are described below.

3,5-Diacetoxybenzoic acid (2): To a solution of 3,5-dihydroxybenzoic acid 1 (0.050 mol, 7.70 g) in pyridine (50 mL), acetanhydride (0.20 mol, 18.9 mL) was added dropwise at 0 °C. After the addition of Ac_2_O, the reaction mixture was stirred for 2 h at room temperature and then slowly poured in water (150 mL). Concentrated HCl was added to adjust the pH to 2 and extracted with ethyl acetate (3 × 150 mL). Combined organic phases were washed with brine (150 mL) and dried with anhydrous sodium sulfate. Sodium sulfate was filtered off and ethyl acetate evaporated under reduced pressure. The solid obtained was suspended in diethyl ether and filtered off to obtain 3.10 g (26%) of 2 as white needles (Mp 144–146 °C (152-153 °C) [30]). ^1^H NMR (400 MHz, DMSO-d_6_): δ 2.29 (s, 6H, 2 × CH_3_), 7.29 (t, 1H, J = 2.2 Hz, Ar-H), 7.58 (d, 2H, J = 2.2 Hz, Ar-H), 13.41 (bs, 1H, COOH) ppm; ESI-HRMS *m/z* calcd. for C_11_H_10_O_6_ [M−H]^−^ 237.0399, found 237.0397; IR (KBr, cm^−1^): 2936, 2597, 1774, 1696, 1603, 1438, 1370, 1309, 1190, 1127, 1025, 913, 892, 838, 774, 754, 704, 660, 591, 520, 460 cm^−1^.

5-((3-Cyano-4,5,6,7-tetrahydrobenzo[*b*]thiophen-2-yl)carbamoyl)-1,3-phenylene diacetate (**3**): To a solution of 3,5-diacetoxybenzoic acid 2 (9.0 mmol, 2.14 g) in anhydrous dichloromethane (40 mL), oxalyl chloride (18.0 mmol, 1.55 mL) was added dropwise at T = 0 °C. After the addition of catalytic amounts of DMF (2–3 droplets) the reaction mixture was stirred for 1 h at room temperature. The solvent was evaporated to dryness to obtain crude acid chloride, which was dissolved in anhydrous dichloromethane (50 mL). The solution of 2-amino-4,5,6,7-tetrahydrobenzo[*b*]thiophene-3-carbonitrile (9.0 mmol, 2.5 g) in anhydrous pyridine (31.5 mmol, 2.6 mL) was added dropwise at T = 0 °C, and the reaction mixture was stirred for 24 h at room temperature. Additional 50 mL of dichloromethane was added and washed with 1 M HCl (50 mL), saturated aqueous solution of NaHCO_3_ (50 mL) and brine (50 mL), dried with anhydrous sodium sulfate, filtered and evaporated under reduced pressure. The crude product was crystallized from ethanol to obtain 1.93 g (54%) of 3 as off-white needles (mp 158–160 °C). ^1^H NMR (400 MHz, DMSO-d_6_): δ 1.74-1.81 (m, 4H, (CH_2_)_2_), 2.32 (s, 6H, 2 × CH_3_), 2.51-2.56 (m, 2H, CH_2_), 2.61-2.66 (m, 2H, CH_2_), 7.33 (t, 1H, J = 2.1 Hz, Ar-H), 7.65 (d, 2H, J = 2.1 Hz, Ar-H), 11.82 (s, 1H, NH) ppm; ^13^C NMR (100 MHz, DMSO-d_6_): δ = 20.83, 21.71, 22.59, 23.50, 23.65, 96.48, 114.14, 119.36, 120.32, 129.10, 131.65, 134.37, 145.76, 150.76, 163.45, 168.98 ppm. ESI-HRMS *m/z* calcd. for C_20_H_18_N_2_O_5_S [M+H]^+^ 399.1015, found 399.1020; IR (KBr, cm^−1^): 3338, 3077, 2940, 2211, 1769, 1679, 1572, 1438, 1400, 1373, 1302, 1196, 1021, 915, 796, 770, 747, 699, 654, 527, 477 cm^−1^.

*N*-(3-Cyano-4,5,6,7-tetrahydrobenzo[*b*]thiophen-2-yl)-3,5-dihydroxybenzamide (SIM-53B): To a stirred suspension of 5-((3-cyano-4,5,6,7-tetrahydrobenzo[*b*]thiophen-2-yl)carbamoyl)-1,3-phenylene diacetate 3 (1.15 mmol, 0.46 g) in methanol (6.0 mL), potassium carbonate (3.45 mmol, 0.48 g) was added and stirred at room temperature for 1 h. The residue obtained after evaporation of methanol was dissolved in 1 M HCl (50 mL) and extracted with ethyl acetate (3 × 50 mL). Combined organic phases were washed with 1 M HCl (30 mL), brine (30 mL) and dried with anhydrous sodium sulfate. Sodium sulfate was filtered off and ethyl acetate evaporated under reduced pressure. The solid obtained was recrystallized from ether and petroleum ether to obtain 0.17 g (47%) of SIM-53B as white needles (mp 260–263 °C). ^1^H NMR (400 MHz, DMSO-d_6_): δ 1.75–1.81 (m, 4H, (CH_2_)_2_), 2.51–2.56 (m, 2H, CH_2_), 2.61-2.66 (m, 2H, CH_2_), 6.46 (t, 1H, J = 2.2 Hz, Ar-H), 6.78 (d, 2H, J = 2.2 Hz, Ar-H), 9.68 (s, 2H, 2 × OH), 11.51 (s, 1H, NH) ppm; ^13^C NMR (100 MHz, DMSO-d_6_): δ = 21.70, 22.58, 23.43, 23.58, 95.79, 106.23, 106.34, 114.22, 128.46, 131.24, 134.28, 146.33, 158.41, 165.21 ppm. ESI-HRMS *m/z* calcd. for C_16_H_14_N_2_O_3_S [M+H]^+^ 315.0803, found 315.0799; IR (KBr, cm^−1^): 3368, 3245, 2928, 2221, 1763, 1654, 1598, 1576, 1560, 1458, 1400, 1312, 1261, 1203, 1173, 1010, 915, 874, 840, 792, 751, 670, 478 cm^−1^. HPLC t_R_ = 12.536 min (97.62% at 254 nm, 97.19% at 220 nm).

### 2.3. Emulsion Preparation

Olive oil-in-water emulsions at different ratios of oil (O) and water (W)—4:6 and 1:9, O/W—were produced by mixing stripped olive oil, acidic water and Tween 20 as the emulsifier. The pH of the aqueous phase was adjusted to 3.65 employing 0.04 M citric acid/citrate buffer. The emulsifier volume fractions Φ_I_ ranging from 0.5% to 4% were used. The mixture was blended for 1 min using a Polytronic PT-1600 homogenizer at room temperature. The required amount of SIM-53B was added to the emulsion at a final concentration of 1 mM.

### 2.4. Antioxidant Activity Determined by DPPH^•^, ABTS and CUPRAC Assays

Free-radical scavenging activity was evaluated employing the DPPH^•^ and ABTS assays [31]. 2,2-Diphenyl-1-picrylhydrazyl radical (DPPH^•^) was dissolved in ethanol (150 μL, 140 μM) and added to 150 μL ethanol solution of the test sample (62.5–500 μM of SIM-53B) or ethanol (negative control) in each well of a flat-bottomed 96-well microliter plate (TPP, Tissue Culture Test Plates). The reaction between DPPH^•^ and SIM-53B was then monitored at λ = 517 nm by using a Synergy H4 Hybrid Multi-Mode Microplate Reader (Bio-Tek Instruments, Inc) at T = 20 °C in the dark for 180 min. Each set of experiments was performed in triplicate. For the reduction kinetics of the DPPH^•^, absorbance was measured using an Agilent Cary 3500 UV-Vis spectrophotometer with the Compact Peltier UV-Vis Module, set at a wavelength of 517 nm. Spectrophotometric disposable (2 mL capacity and 1 cm path-length) cuvettes were used. The absorbance of a sample and a blank were measured simultaneously at a sampling rate of one point per second. Automatic acquisition of data was set for a reaction time of 90 min 5 s after mixing DPPH^•^ (70 μM) with SIM-53B solution in methanol. All samples were analyzed in duplicate at temperature (20.0 ± 0.1) °C.

For the ABTS assay, a slightly modified procedure described in [32] was used. To 10 mL of 7 mM stock solution of 2,2′-azinobis(3-ethylbenzothiazoline-6-sulfonic acid ammonium salt) (ABTS) was added 178 µL 140 mM solution of potassium persulfate. Working solution was allowed to react for 16 h at room temperature in the dark. The solution was than diluted by mixing 1 mL ABTS^•+^ with 64 mL of ethanol (96%) to obtain an absorbance of 1.1 units at 734 nm. The solution of SIM-53B and solutions of standards (Trolox and resveratrol) were freshly prepared in 96% ethanol at 1 mM concentration. To a test tube were added ethanol [(2.00−x) mL] and solution of SIM-53 (or standard) (x mL) and finally 2 mL of ABTS^•+^ so as to make the final volume 4.0 mL. The tubes were closed by parafilm, and the mixtures were vortexed and incubated for 90 min at room temperature in a dark condition. Absorbance at λ = 734 nm was recorded against a blank using the mentioned UV–Vis spectrophotometer. The calibration curve obtained can be found in the Supplementary material.

Trolox equivalent antioxidant capacity (TEAC_CUPRAC_) of SIM-53B was determined using its Cu^2+^ reducing capability in the presence on neocuproine by the CUPRAC method [33]. The solution of SIM-53B and solutions of standards (Trolox and resveratrol) were freshly prepared in 96% ethanol at 1 mM concentration. To a test tube were added 1 mL each of CuCl_2_ (10 mM in water), neocuproine (7.5 mM in 96% ethanol) and ammonium acetate buffer (pH 7, 1 mM in water) solutions. SIM-53 (or standard) solution (x mL) and water [(1.10−x) mL] were added to the mixture so as to make the final volume 4.1 mL. The tubes were closed by parafilm, and the mixtures were vortexed and incubated for 60 min at room temperature (or in a water bath at T = 50 °C for 20 min). Absorbance at 450 nm was recorded against a reagent blank using the mentioned UV–Vis spectrophotometer. The molar absorptivity (ε) for each antioxidant was calculated from the slope of the calibration line by plotting absorbance versus concentration (the calibration curve obtained can be found in the Supplementary material). TEAC_CUPRAC_ was calculated by dividing the molar absorptivity of SIM-53B or resveratrol by that of Trolox.

### 2.5. Pseudophase Kinetic Model Applied to Emulsions: Partition Constants and Distribution of SIM-53B

In an emulsified system, SIM-53B can be transferred between the oil, aqueous and interfacial regions, Scheme 1. Thus, the distribution of SIM-53B can be defined by two partition constants, that between the aqueous and interfacial regions, *P*_W_^I^ (Equation (1)), and that between the oil and interfacial regions, *P*_O_^I^ (Equation (2)). The partition constants were assessed in the intact emulsions by making use of a chemical kinetic method that grounded the reaction between a chemical probe (4-hexadecylbenzenediazonium ion, 16-ArN_2_^+^) and SIM-53B in the interfacial region and the experimental data were interpreted on the basis of the pseudophase kinetic model. In brief, the suppositions of the pseudophase kinetic model are that the partitioning of a compound is in dynamic equilibrium in an emulsified system and it depends on its relative solubility in the aqueous, oil and interfacial regions. Details of the method can be found elsewhere [22].

(1) $$P_{W}^{I}=\frac{{(\mathrm{SIM}}_{I})}{{(\mathrm{SIM}}_{W})}$$

(2) $$P_{O}^{I}=\frac{{(\mathrm{SIM}}_{I})}{{(\mathrm{SIM}}_{O})}$$

Equation (3) has been derived elsewhere [22] and describes the relationship between the observed rate constant *k*_obs_ for the reaction between 16-ArN_2_^+^ and SIM-53B and the partition constants, *P*_O_^I^ and *P*_W_^I^. Equation (3) predicts that *k*_obs_ values should decrease upon increasing Φ_I_ because the stoichiometric SIM-53B concentration ([SIM_T_]), Φw (volume fraction of water) and Φo (volume fraction of oil) are constant in the kinetic experiments.

Equation (4) is equivalent to Equation (3) and the parameters a and b are defined by Equations (5) and (6), respectively. The parameters *a* and *b* can be determined by linear least squares fitting of the (1/*k*_obs_, Φ_I_) pairs of data, Equation (7). Therefore, *P*_O_^I^ and *P*_W_^I^ values can be calculated from the variations in *k*_obs_ with Φ_I_ in two set of kinetics experiments for two different oil-in-water ratios by solving a system of two equations and two unknowns (Equation (6) for two different Φ_O_ and Φ_W_ values).

(3) $$k_{obs}=k_{I}\left(SIM_{I}\right)=\frac{\left[SIM_{T}\right]k_{I}P_{W}^{I}P_{O}^{I}}{\Phi_{O}P_{W}^{I}+\Phi_{{}_{I}}P_{W}^{I}P_{O}^{I}+\Phi_{W}P_{O}^{I}}$$

(4) $$k_{obs}=\frac{a}{1+b\Phi_{I}}$$

(5) $$a=\frac{{\left[SIM\right]}_{T}k_{I}P_{W}^{I}P_{O}^{I}\left(1+\Phi_{W}/\Phi_{O}\right)}{P_{W}^{I}+\Phi_{W}/\Phi_{O}P_{O}^{I}}$$

(6) $$b=\frac{P_{W}^{I}P_{O}^{I}\left(1+\Phi_{W}/\Phi_{O}\right)}{P_{W}^{I}+\Phi_{W}/\Phi_{O}P_{O}^{I}}-1$$

(7) $$\frac{1}{k_{obs}}=\frac{1}{a}+\frac{b}{a}\Phi_{I}$$

Once the partition constants are known, the percentages of SIM-53B in the different regions of the emulsion are obtained by using Equations (8–10). Details of the calculations are provided elsewhere [22].

(8) $$\%SIM_{I}=\frac{100\Phi_{I}P_{W}^{I}P_{O}^{I}}{\Phi_{O}P_{W}^{I}+\Phi_{{}_{I}}P_{W}^{I}P_{O}^{I}+\Phi_{W}P_{O}^{I}}$$

(9) $$\%SIM_{W}=\frac{100\Phi_{W}P_{O}^{I}}{\Phi_{O}P_{W}^{I}+\Phi_{{}_{I}}P_{W}^{I}P_{O}^{I}+\Phi_{W}P_{O}^{I}}$$

(10) $$\%SIM_{O}=\frac{100\Phi_{O}P_{W}^{I}}{\Phi_{O}P_{W}^{I}+\Phi_{{}_{I}}P_{W}^{I}P_{O}^{I}+\Phi_{W}P_{O}^{I}}$$

### 2.6. Determination of k_obs_ in Opaque Emulsion by Spectrophotometry

The reaction between 16-ArN_2_^+^ and SIM-53B was followed spectrophotometrically in opaque olive oil-in-water emulsions by employing a colorimetric assay (derivatization reaction) described in detail elsewhere [34]. The derivatization reaction was grounded in the speedy reaction between the chemical probe, 16-ArN_2_^+^ ions, and an appropriate coupling agent (N-(1-naphthyl)ethylenediamine dihydrochloride, NED) producing an instant and stable azo dye.

In a common experiment, a SIM-53B-loaded emulsion was located in a stirred and thermostated (T = 25 °C) water-jacketed cell. Aliquots of 200 µL of emulsion were removed at selected time intervals and added to 15 test tubes each containing 2.5 mL of the reagent solution (alcoholic NED solution 19 mM, 50:50 (v:v) BuOH/EtOH). The optimal absorption wavelength was found to be 572 nm and was selected for azo dye determination. Auxiliary experiments confirmed that the absorption of the azo dye was proportional to the concentration of 16-ArN_2_^+^ ions that do not react with SIM-53B. The absorbance values obtained were plotted against the time and the observed first order rate constants, *k*_obs_, were determined from the fitting absorbance—time data to the integrated first order rate equation (Equation (11)), where A_t_, A_o_ and A_∞_ are the measured absorbance at any time, at t = 0 and at infinite time, respectively [34].

(11) $$\ln\frac{A_{t}-A_{\infty }}{A_{0}-A_{\infty }}=-k_{obs}t$$

### 2.7. Statistical Analysis

**Figure 1:**
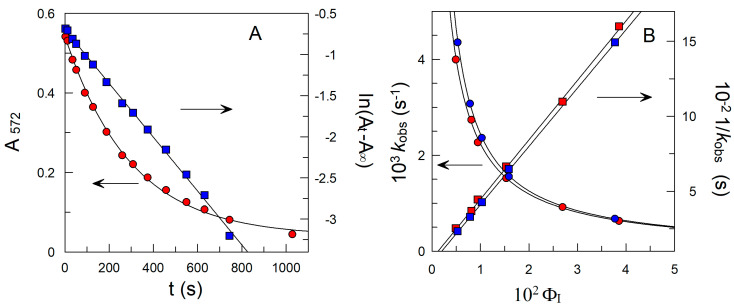
(**A**) Changes in the absorbance (-●-, λ = 572 nm) of the formed azo dye with the time and ln(A_t_–A_∞_) vs. time plot (-■-). Reaction conditions: olive oil-in-water 1:9 (O/W) emulsions, Φ_I_ (Tween 20) = 0.0079, pH of the aqueous phase was adjusted to 3.65 employing citric acid/citrate buffer 0.04 M, [16-ArN_2_^+^] = 0.29 mM, [SIM-53B] = 1 mM, T = 25 °C. The absorbance of azo dye is proportional to the unreacted [16-ArN_2_^+^] [34]. (**B**) Variations in the observed rate constants (*k*_obs_) with the emulsifier volume fraction (Φ_I_) for olive oil-in-water emulsions 1:9 (-●-,-■-, O/W) and 4:6 (-●-,-■-, O/W). Solid lines are the theoretical curves obtained by fitting the experimental data to the equation derived from the pseudophase kinetic model (Equation (4)) and to its reciprocal, Equation (7).

Each set of kinetic experiments was performed in triplicate giving observed rate constants *k*_obs_ values within ± 8%. Uncertainty in the *P*_O_^I^ and *P*_W_^I^ values was calculated by propagation of the standard error of the slope and intercept of the equation fit of the linear plots illustrated in Figure 1. In all cases, results are expressed as average ± standard deviation (*n* = 3).

## 3. Results and Discussion

### 3.1. Partition Constants Values P_W_^I^ and P_O_^I^ for SIM-53B

Figure 1 shows a typical kinetic plot illustrating the changes in the absorbance of the azo dye with time (circles) and the fitting curve to the integrated and linearized first-order equation (Equation (11), squares) for the reaction between 16-ArN_2_^+^ and SIM-53B in 1:9 (O/W) olive oil-in-water emulsions. The slopes of the linear fits of ln (A_t_ - A_∞_) vs. time were used to calculate the *k_obs_* values (Figure 1B). Figure 1B illustrates the variations in *k*_obs_ with Φ_I_ for SIM-53B in 1:9 and 4:6 (O/W) olive oil-in water emulsions. In both kinetics experiments, *k*_obs_ decreases asymptotically with increasing Φ_I_ by a factor ~6 on going from Φ_I_ = 0.005 to Φ_I_ = 0.04 for both emulsions. The excellent fits of Equations (4) and (7) to the *k*_obs_ vs. Φ_I_ plots and their reciprocals in Figure 1B show that the hypotheses considered in the derivation of Equation (3) are met [22]. The partition constants *P*_W_^I^ and *P*_O_^I^ and the *k*_I_ values were determined from the slopes and intercepts of the linear fits of plots of 1/*k*_obs_ vs. Φ_I_ and values are included in Table 1.

An initial analysis of the *P*_W_^I^ and *P*_O_^I^ values obtained for SIM-53B, Table 1, indicates that both values are quite high and positive, suggesting the tendency of SIM-53B to be distributed in the interfacial region of the olive oil-in-water emulsions. This fact means that the processes of transfer of SIM-53B from the oil to the interfacial and from the aqueous to the interfacial regions are spontaneous, with *P*_W_^I^ >>> *P*_O_^I^ reflecting the relative higher solubility of SIM-53B in olive oil than in the aqueous solution.

For comparative purposes, note that the value of the partition constant between the oil and aqueous phase in the absence of an emulsifier, *P*_W_^O^, for SIM-53B can be defined as the ratio between the *P*_W_^I^ and *P*_O_^I^ values and calculated from Equation (12). A value of *P*_W_^O^ = 8.6 ± 1.6 (Log *P*_W_^O^ = 0.9 ± 0.2) was calculated at T = 25 °C, indicating its higher oil solubility in keeping with its hydrophobic nature. Note that the Log *P*_W_^O^ value is ~2.9 times lower than that calculated in n-octanol/water mixtures (Log *P*_W_^OCT^, Table 1), reflecting that SIM-53B is less soluble in olive oil than in octanol, questioning if the octanol/water system is the best model system for analyzing the hydrophobic effect in more complex biologic systems.

(12) $$P_{W}^{O}=\frac{{(\mathrm{SIM}}_{O})}{{(\mathrm{SIM}}_{W})}=\frac{P_{W}^{I}}{P_{O}^{I}}$$

In parallel, the rate constant for the reaction between the chemical probe and SIM-53B, *k*_I_, was also determined (Table 1). This value is not necessary to describe how SIM-53B partitions between the different regions but its knowledge may be important because it delivers insights into the aspects of the reaction mechanism for the reaction between the chemical probe and SIM-53B. The *k*_I_ value is comparable to that of the value obtained for chlorogenic acid (*k*_I_ = 15 mM^−1^s^−1^) [26] but lower for that of, for example, gallic acid (*k*_I_ = 53 mM^−1^s^−1^) [36], hydroxytyrosol (*k*_I_ = 117 mM^−1^s^−1^) [29] or α-tocopherol (*k*_I_ = 190 mM^−1^s^−1^) (unpublished results).

### 3.2. Distribution of SIM-53B and Its Local Effective Concentrations: Effect of Emulsifier Volume Fraction and O/W Ratio

**Figure 2:**
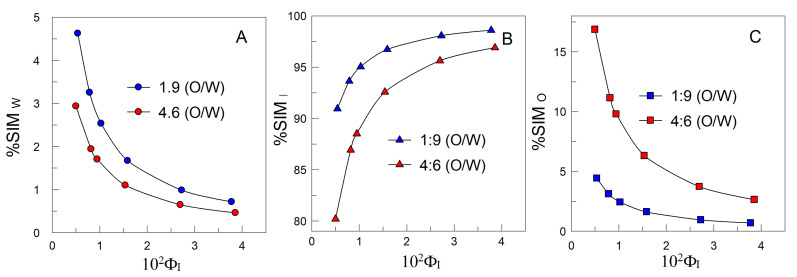
Effect of emulsifier volume fraction (Φ_I_) on the percentages of SIM-53B in the aqueous (**A**), in the interfacial (**B**) and in the oil (**C**) regions of 1:9 and 4.6 (O/W) emulsions.

Once partition constants *P*_O_^I^ and *P*_W_^I^ were known, the percentage of SIM-53B in each region of the olive oil-in-water emulsions was determined by employing Equations (8–10) in order to analyze its availability in the different regions of the emulsions, Figure 2. Details of the determination of the percentages of SIM-53B are given in Section 2.5.

Results in Figure 2 show that the percentage of SIM-53B in the different regions depends on both the emulsifier volume fraction (Φ_I_) and on the O/W ratio. At a given O/W ratio, results indicate that SIM-53B is primarily distributed in the interfacial region of the olive oil-in-water emulsions. A percentage of SIM-53B in the interfacial region, higher than ~80% (Φ_I_ = 0.005, 4:6, O/W) is located in this region with percentages lower than ~17% (Φ_I_ = 0.005, 4:6, O/W) and ~3% (Φ_I_ = 0.005, 4:6, O/W) in the oil and aqueous regions, respectively. For instance, the interfacial percentage of SIM-53B (%SIM _I_) increases from 80% to 96% (4:6, O/W) and from 91% to 98% (1:9, O/W) by increasing the interfacial volume ~7 times (from Φ_I_ = 0.005 up to Φ_I_ = 0.035), with the corresponding decrease in the oil and aqueous regions.

At a given Φ_I_, the %SIM _I_ increases upon increasing the O/W ratio used in the preparation of the emulsion. For example, the %SIM_I_ increases from 80% (Φ_I_ = 0.005, 4:6, O/W) to 91% (Φ_I_ = 0.005, 1:9, O/W) and the differences are less significant at high Φ_I_ values than at low Φ_I_ values.

**Figure 3:**
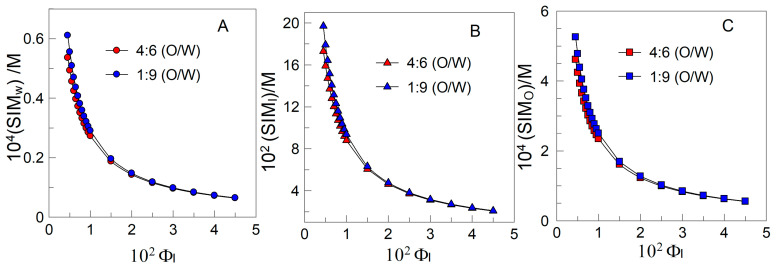
Effect of the emulsifier volume fraction (Φ_I_) on the effective local concentrations of SIM-53B in the aqueous (**A**), in the interfacial (**B**) and in the oil (**C**) regions of 1:9 (O/W) and 4:6 (O/W) olive oil-in-water emulsions. The concentrations given in parenthesis refer to the moles of SIM-53B per liter of the aqueous (W), oil (O) and interfacial (I) regions. Added SIM-53B concentration, [SIM_T_] = 1 mM.

The effective local concentration of SIM-53B in the different regions of the olive oil-in-water emulsions (Figure 3) can be determined by employing Equations (13)–(15) where [SIM_T_] stands for the stoichiometric concentration of SIM-53B ([SIM_T_] = 1 mM) and Φ_W_, Φ_I_ and Φ_O_ refer to the water, interfacial and oil volume fractions, respectively.

(13) $$(SIM_{W})=\frac{\left[SIM_{T}\right]\left(\%SIM_{W}\right)}{\Phi_{W}}$$

(14) $$(SIM_{I})=\frac{\left[SIM_{T}\right]\left(\%SIM_{I}\right)}{\Phi_{I}}$$

(15) $$(SIM_{O})=\frac{\left[SIM_{T}\right]\left(\%SIM_{O}\right)}{\Phi_{O}}$$

Results in Figure 3 highlight three relevant points. First, from the comparison of the results in Figure 2, Figure 3, it can be seen that the (%SIM_I_) increases upon increasing Φ_I_ (Figure 2), whereas it has the opposite effect on the interfacial concentrations of SIM-53B (Figure 3). This fact can be explained on the basis of the Equations (13)–(15). As described in Equation (14), the interfacial concentration of SIM-53B depends not only on the percentage of SIM-53B in the interfacial region but also on the interfacial volume. In this particular case, the increase in the incorporation of SIM-53B to the interfacial region of the emulsion does not compensate the increase in the interfacial volume resulting in the corresponding decrease in the interfacial concentration of SIM-53B. For example, the percentage of SIM-53B in the interfacial region only increases ~1.1-fold from %SIM_I_ = 80 (Φ_I_ = 0.005, 4:6 O/W) to %SIM_I_ = 96 (Φ_I_ = 0.035, 4:6 O/W) upon increasing the interfacial volume ~7-fold, leading to a decrease in (SIM_I_) from 0.17 (Φ_I_ = 0.005, 4:6 O/W) to 0.02 M (Φ_I_ = 0.035, 4:6 O/W) according to Equation (14).

Second, the interfacial concentrations of SIM-53B are ~ 20–200 times (depending on the Φ_I_ value) higher than the stoichiometric concentration of SIM-53B ([SIM_T_] = 1 mM), while the concentrations of SIM-53B in the oil and aqueous regions become much smaller than the stoichiometric concentration of SIM-53B. Notice that this observation may produce a significant change in its potential biological activity by amplifying it since the rate of any reaction depends on the concentrations of reagents at the reaction site.

Third, changes in the local concentrations of SIM-53B with O/W ratio from 1:9 to 4:6 (O/W) used in the preparation of the emulsion are not significant. For example, at Φ_I_ = 0.005, (SIM_I_) changes from 0.17 to 0.2 M upon decreasing the O/W ratio (differences less than 15%), and the differences are much less at higher emulsifier volume fractions (Φ_I_ = 0.045) than at lower emulsifier volume fractions (Φ_I_ = 0.005). Therefore, changes in the oil-in-water ratio should have predictably an almost negligible effect on the antioxidant efficiency of SIM-53B in inhibiting lipid oxidation since the effectiveness of the series of homologous AOs is directly related to their distribution according to our previous work [25,26,27,28,29].

### 3.3. Radical Scavenging Activity of SIM-53B

The antioxidant efficiency of a compound to inhibit oxidative damage caused by free radicals depends not only on its concentration at the reaction site but also its reactivity against free radicals. The radical scavenging activity of SIM-53B was assessed by employing the stable free radical 2,2-diphenyl-1-pycrilhydrazyl (DPPH^•^) and 2,2′-azinobis(3-ethylbenzothiazoline-6-sulfonic acid) (ABTS). Antioxidant capacity was expressed in terms of the EC_50_ value, labelled as the concentration of SIM-53B needed to decrease the DPPH^•^ or ABTS^•*+*^ radical concentration by 50%.

**Figure 4:**
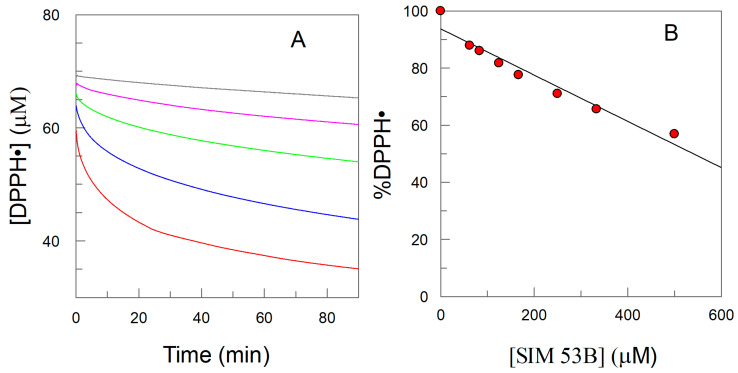
(**A**) Time course for the variation in the DPPH^•^ concentration ([DPPH^•^] _initial_ = 70 μM) in the presence of different concentrations of SIM-53B (**---** 35 μM**, ---** 70 μM, **---** 140 μM, **---** 350 μM, **---** 700 μM) at T = 20 °C. (**B**) Changes in the percentage of the initial DPPH^•^ radical remaining (%DPPH^•^) after the reaction with seven different concentrations of SIM-53B (62.5–500 μM of SIM-53B) at time 180 min and T = 20 °C.

Figure 4A shows a typical kinetic plot obtained for the variation in the concentration of the DPPH^•^ radical in the presence of different concentrations of SIM-53B at T = 20 °C. The percentage of the remaining DPPH^•^ at a certain time of the reaction was obtained by using Equation (16), where A_0_ is the absorbance of the negative control and A_1_ is the absorbance of the test sample, and it was plotted against the concentration of SIM-53B (Figure 4B). Trolox and resveratrol were used as positive controls in this assay with EC_50_ 12.4 ± 0.3 and 50.1 ± 4.6 μM, respectively [31]. The EC_50_ value obtained for SIM-53B is listed in Table 1.

(16) $$DPPH^{•}(\%)=\frac{A_{0}-A_{1}}{A_{0}}\cdot 100$$

The EC_50_ value determined by the DPPH method for SIM-53B (440 μM, Table 1) is around 80 times higher than that observed for phenolic compounds such as gallic acid (3,4,5-trihidroxybenzoic acid, EC_50_ = 5.1 μM [37]) or catechin (EC_50_ = 6 μM [37]), suggesting a much lower reactivity of resorcinol derivatives vs. catechols towards DPPH^•^ radicals. This can be explained by the presence of a second hydroxyl group in the meta position which enables intramolecular hydrogen bonding and more potent antioxidant activity [38,39]. Indeed, the antiradical efficiency of dihydroxybenzenes was determined in the following order: catechol > hydroquinone > resorcinol [40]. Furthermore, a similar pattern was observed in the series of dihydroxybenzaldehydes, where 2,3 or 3,4-dihydroxybenzaldehyde exhibited the strongest antioxidant activities (EC_50_ between 2.3 and 15.7 μM in the ABTS and DPPH^•^ assays), whereas 3,5-dihydroxybenzaldehyde was less potent in antioxidant activities with an EC_50_ of 20.59 and >100 μM in the ABTS and DPPH^•^ assays, respectively [41]. The reduction kinetics of DPPH^•^ caused by SIM-53B are typical for slow-kinetics polyphenols like the flavanone hesperetin [42]. On the contrary to the DPPH method, the EC_50_ value determined by the ABTS method (Supplementary material, Figure S10) for SIM-53B (6.45 ± 0,60 μM, Table 1) is between those of Trolox (17.55 ± 0.36 µM) and resveratrol (5.69 ± 0.47 µM). The Trolox equivalent antioxidant capacity (TEAC_CUPRAC_) of SIM-53B (1.04 at r.t., 1.81 at 50 °C) and resveratrol (1.55 at r.t., 2.31 at 50 °C) determined by the CUPRAC method (Supplementary material, Figure S11) reflects the number of phenolic -OH groups in a molecule and depends on temperature which is again typical for slow-kinetics polyphenols [33]

### 3.4. Prediction of Dug-Like Properties Pharmacokinetic Profile

The pharmacokinetic profile of SIM-53B was evaluated by employing the SwissADME (absorption, distribution, metabolism and excretion of drugs) tool, a computer-designed model based on Lipinski’s rule of five [43,44]. This rule states that high absorption or permeation of a compound is more probable when its chemical structure fulfils two or more of the following conditions: molecular weight is less than 500 Da; no more than five hydrogen bond donors (-NH-, -OH); no more than ten hydrogen bond acceptors (-N=, -O-); and octanol–water partition coefficient not greater than 5. This study was carried out to forecast the pharmacokinetic profile, potential biological activities and toxicity of SIM-53B before it is assessed as a potential oral active drug [45]. Poor pharmacokinetic profile and toxicity are the main reasons for failures in drug discovery. Some parameters such as blood–brain barrier (BBB) permeation, human drug-likeness, interaction with cytochrome P450 (isoenzymes that play an important role in drug elimination through metabolic transformation) and bioavailability score were predicted, Table 1 [43].

The calculated “drug-likeness” value and the Abbot bioavailability score (defined as the probability of a compound to have at least 10% oral bioavailability in rat or measurable Caco-3 permeability, respectively) predict that SIM-53B may become a potential oral drug candidate since no violations to the Lipinski’s rule of five were found, Table 1 [43].

Values predict a high probability of passive absorption by the gastrointestinal tract but not brain penetration. The interaction of SIM-53B with cytochrome P450 isoforms plays an important role in SIM-53B elimination through metabolic biotransformation. SIM-53B may inhibit some of the cytochrome P450 isoforms such as CYP1A2, CYP2C9 and CYP3A4. SIM-53B is not a substrate for p-glycoprotein (P-gp), a protein located in the intestinal epithelium where it pumps drugs back into the intestinal lumen and in the capillary endothelial cells composing the blood–brain barrier where it pumps them back into the capillaries. The skin permeate value suggests that SIM-53B would have low skin permeability.

## 4. Conclusions

We describe the synthesis of a new cyanothiophene-based compound, *N*-(3-cyano-4,5,6,7-tetrahydrobenzo[*b*]thiophen-2-yl)-3,5-dihydroxybenzamide (SIM-53B), and evaluation of its antioxidant activity and distribution in intact stripped olive oil-in-water emulsions. The radical scavenging activity and reductive properties of SIM-53B were assessed by DPPH, ABTS and CUPRAC assays. The reduction kinetics of DPPH^•^ caused by SIM-53B are typical for slow-kinetics polyphenols. Regarding the distribution, results show that SIM-53B has a natural tendency to be incorporated into the interfacial region of olive oil-in-water emulsions and its interfacial concentration is much higher (~20–200-fold) than the total added concentration due to the smaller interfacial volume in comparison with that of the total volume of the emulsion. The interfacial percentage of SIM-53B depends on both the O/W ratio and the emulsifier volume fraction (Φ_I_). An increase in the emulsifier volume fraction increases its percentage from 91% (Φ_I_ = 0.005) to 98% (Φ_I_ = 0.04) for 1:9 (O/W) emulsions but decreases its interfacial concentration ~9 times as a consequence of the higher increase in the interfacial volume than in the percentage of SIM-53B in the interface. Changing the O/W ratio from 4:6 (O/W) to 1:9 (O/W) has a minor effect on the interfacial concentration of SIM-53B due to its high accumulation in the interface at any Φ_I_ value. Taken together, results show that the key parameter that controls the distribution of SIM-53B is the emulsifier volume fraction, suggesting that emulsions with low emulsifier volume fractions may enhance its bioavailability to attain the targets sites.

In addition, the pharmacokinetic properties predicted for this compound are in accordance with the general requirements for potential drugs and its interfacial location makes this new compound a very interesting scaffold for the development of new synthetic compounds that may be effective candidates for preventing or inhibiting oxidation in biomembranes or in other types of lipidic systems.

## Figures and Tables

**Table 1:** *P*_O_^I^ and *P*_W_^I^ and *k*_I_ values in olive oil-in-water emulsions. EC_50_ (μM) values, TEAC_cuprac_ and other Swiss ADME pharmacokinetics parameters. *P*_W_^I^ = partition constant between the aqueous and interfacial region, *P*_O_^I^ = partition constant between the oil and interfacial region, *k*_I_ = rate constant for the reaction between the chemical probe (hexadecylbenzenediazonium ion, 16ArN_2_^+^) and SIM-53B in the interfacial reaction of the O/W emulsion.GI = gastro-intestinal absorption, BBB = blood–brain barrier, CYP = cytochromes, P-pg = P-glycoprotein.

***P*_W_^I^**	3227 ± 426
***P*_O_^I^**	375 ± 49
***k_I_* (M^−1^s^−1^)**	0.0252 ± 0.0077
**EC_50_(DPPH) (μM)**	440.8 ± 48.9
**EC_50_(ABTS) (μM)**	6.45 ± 0.60
**TEAC_cuprac_**	1.04 (rt); 1.81 (50 °C)
Log *P*_W_^OCT^ *	2.73
Solubility *	Moderately soluble
Drug likeness *	Yes
Bioavailability score *	0.55
GI Absorption *	High
BBB permeation *	No
Pg-p substrate *	No
CYP1A2 Inhibitor *	Yes
CYP2C19 Inhibitor *	Yes
CYP2C9 Inhibitor *	Yes
CYP2D6 Inhibitor *	No
CYP3A4 Inhibitor *	Yes
Log *k*p (skin permeation) *	−5.69 cm/s

* Data were obtained from [35].

## Supplementary Materials

The following are available online at https://www.mdpi.com/2076-3921/9/7/623/s1, Figure S1: IR spectrum for 2, Figure S2: ^1^H NMR spectrum for 2, Figure S3: IR spectrum for 3, Figure S4: ^1^H NMR spectrum for 3, Figure S5: ^13^C NMR spectrum for 3, Figure S6: IR spectrum for SIM-53B, Figure S7: ^1^H NMR spectrum for SIM-53B, Figure S8: ^13^C NMR spectrum for SIM-53B, Figure S9: HPLC chromatogram and area percent report for SIM-53B, Figure S10: Antioxidant activity determined by ABTS: calibration curve. Changes of the absorbance of initial ABTS^•^^+^ radical remaining after the reaction with seven different concentrations of SIM-53B (1.25–8.75 μM of SIM-53B) at time 90 min and room temperature., Figure S11: CUPRAC assay: calibration curves. Calibration curves employed to determine TEAC_CUPRAC_ values at room temperature (A and B) and at T = 50 ºC (C and D).
