# Supplementary material for: Synthesis, In Vitro Antioxidant Properties and Distribution of a New Cyanothiophene-Based Phenolic Compound in Olive Oil-In-Water Emulsions
Source: Antioxidants (Basel). 2020 Jul 16;9(7):623. doi: 10.3390/antiox9070623 (PMC7402159; doi:10.3390/antiox9070623)
Supplement: Supplementary file 1 [file antioxidants-09-00623-s001.pdf]

SUPPLEMENTARY MATERIAL

Synthesis, *In Vitro* Antioxidant Properties and Distribution of a New Cyanothiophene-Based Phenolic Compound in Olive Oil-In-Water Emulsions

Sonia Losada-Barreiro[1,2*], Matej Sova[3*], Janez Mravljak[3], Luciano Saso[4], Carlos Bravo-Díaz[1]

[1] Physical Chemistry Department, Chemistry Faculty, University of Vigo E-36310, Spain

[2] REQUIMTE-LAQV, Chemistry and Biochemistry Department, Science Faculty, University of Porto, Porto, PT-4169-007, Portugal.

[3] Faculty of Pharmacy, University of Ljubljana, Aškerčeva 7, 1000 Ljubjana, Slovenia

[4] Department of Physiology and Pharmacology "Vittorio Erspamer", Sapienza University of Rome, Rome, 00185, Italy

*Correspondence: sonia@uvigo.es , Matej.Sova@ffa.uni-lj.si

Table of contents

Figure S1. IR spectrum for 2……………..……………………………………………………3

Figure S2. ^{1}H NMR spectrum for 2………………………..…………...………………..4

Figure S3. IR spectrum for 3…………………………………………………………..……5

Figure S4. ^{1}H NMR spectrum for 3……………………………...…………..……………..6

Figure S5. ^{13}C NMR spectrum for 3……..……………….………………..……………7

Figure S6. IR spectrum for SIM-53B……………………………………..…...…………8

Figure S7. ^{1}H NMR spectrum for SIM-53B……………………………………………..9

Figure S8. ^{13}C NMR spectrum for SIM-53B…………………….…………………..…10

Figure S9. HPLC chromatogram and area percent report for SIM-53B…………….……11-12

Figure S10. Antioxidant activity determined by ABTS: calibration curve ………...………..13

Figure S11. CUPRAC assay: calibration curves ……………………..……………….……14

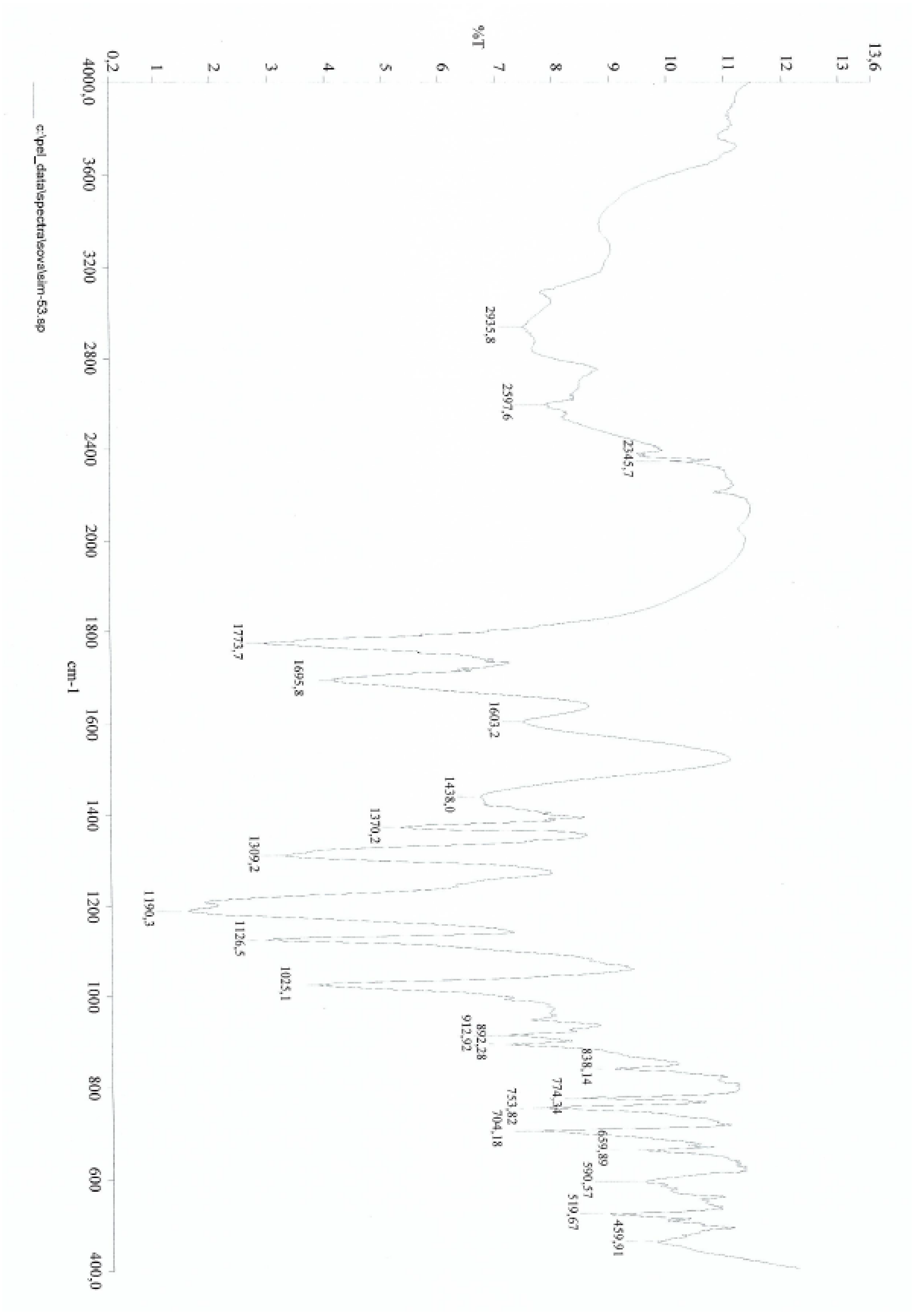

Figure S1. IR spectrum for compound 2.

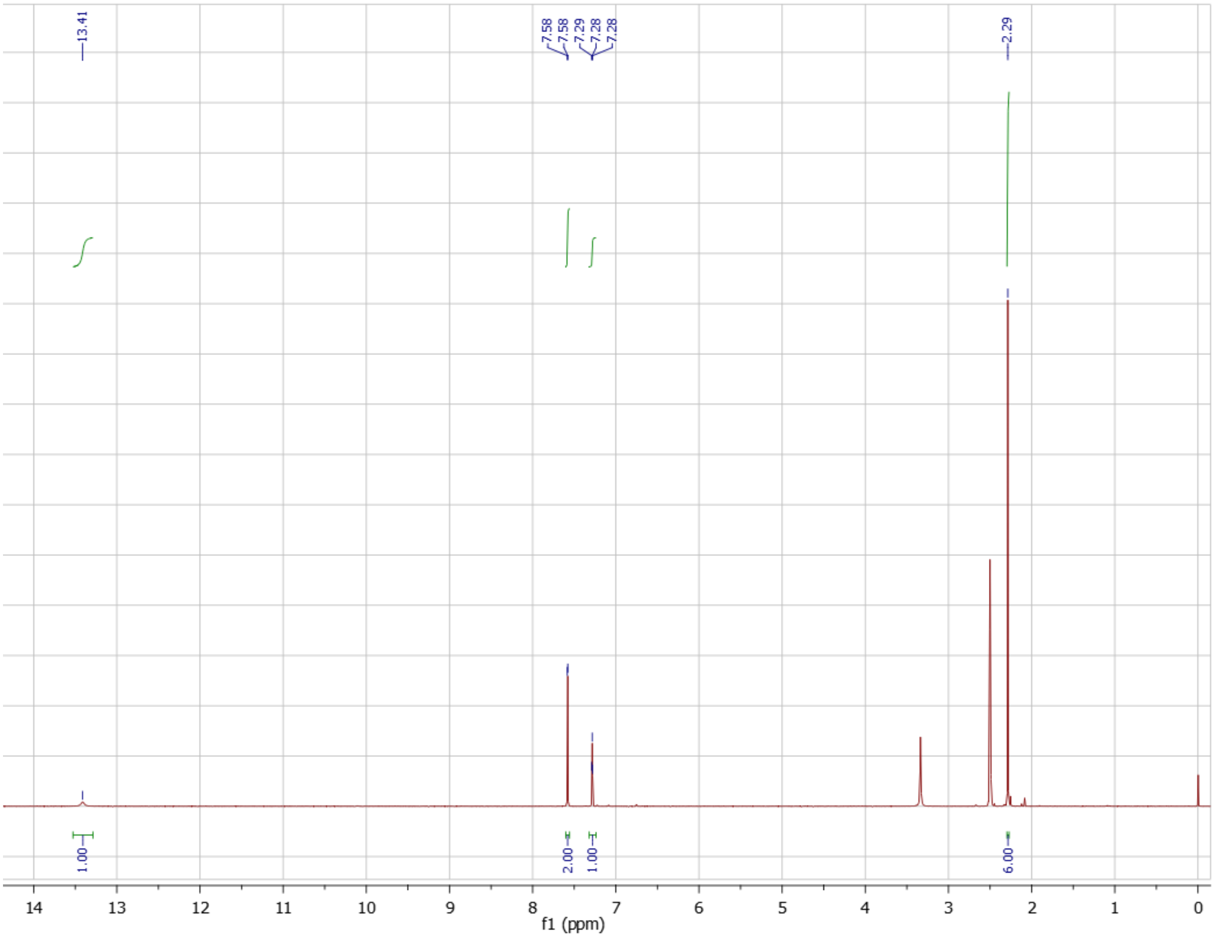

Figure S2. ^{1}H NMR spectrum for compound 2.

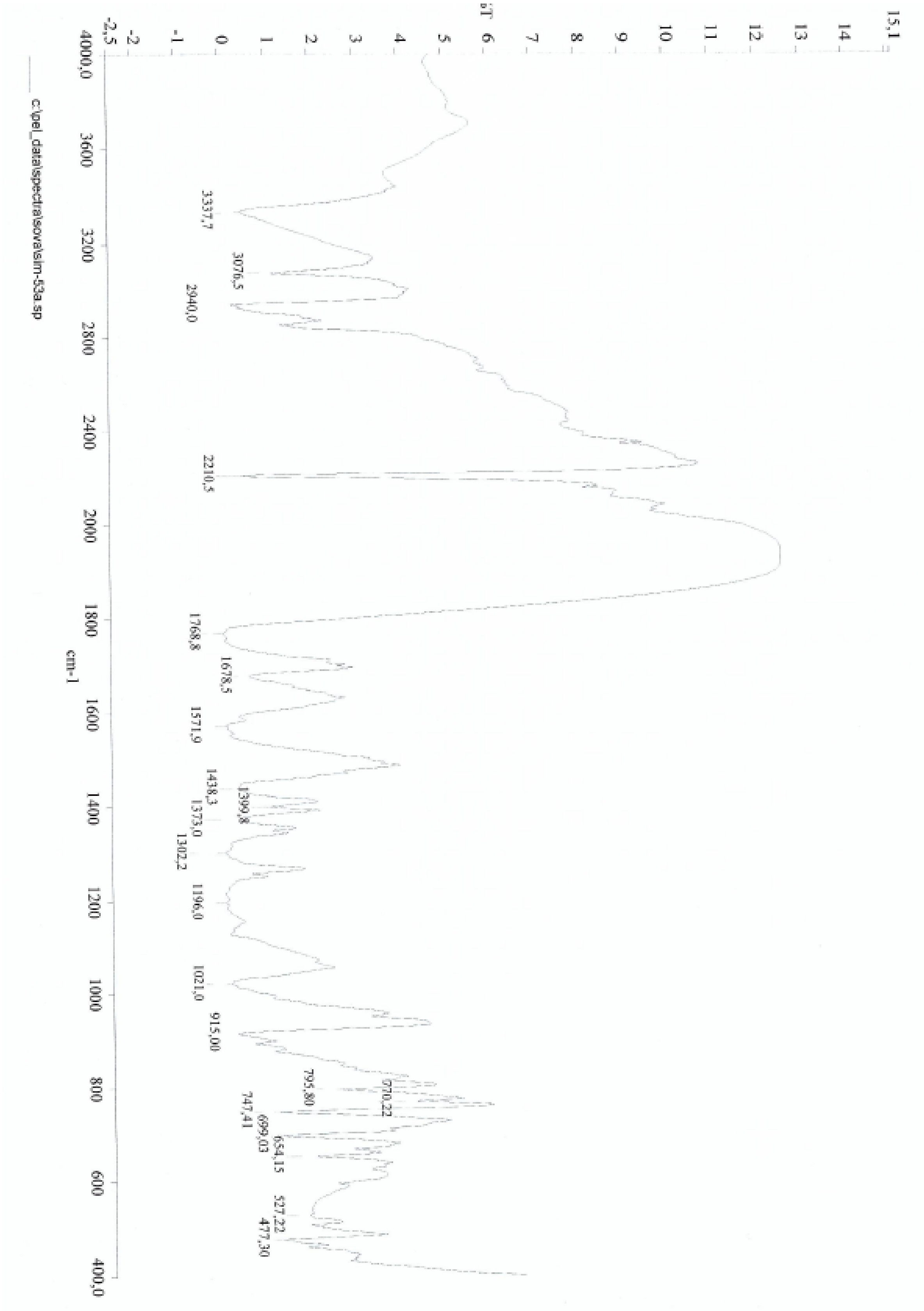

Figure S3. IR spectrum for compound 3.

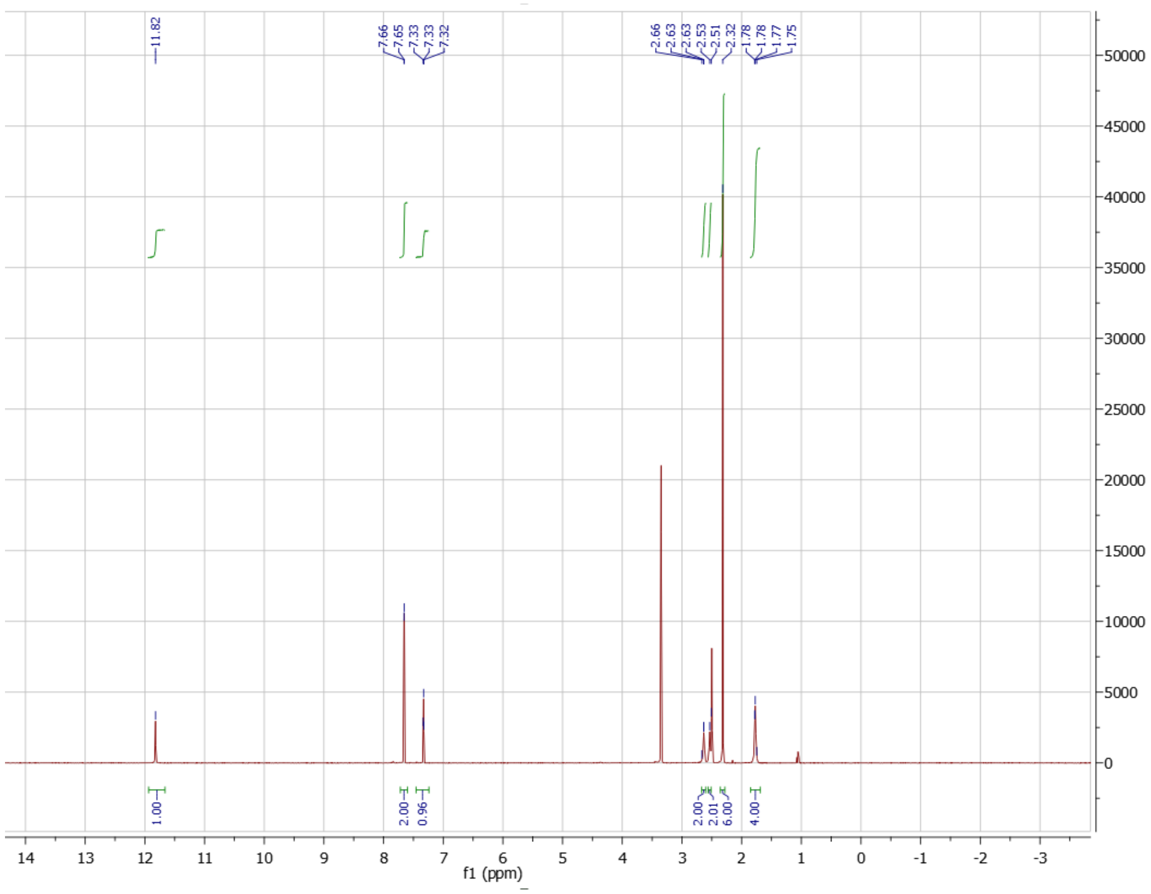

Figure S4. ^{1}H NMR spectrum for compound 3.

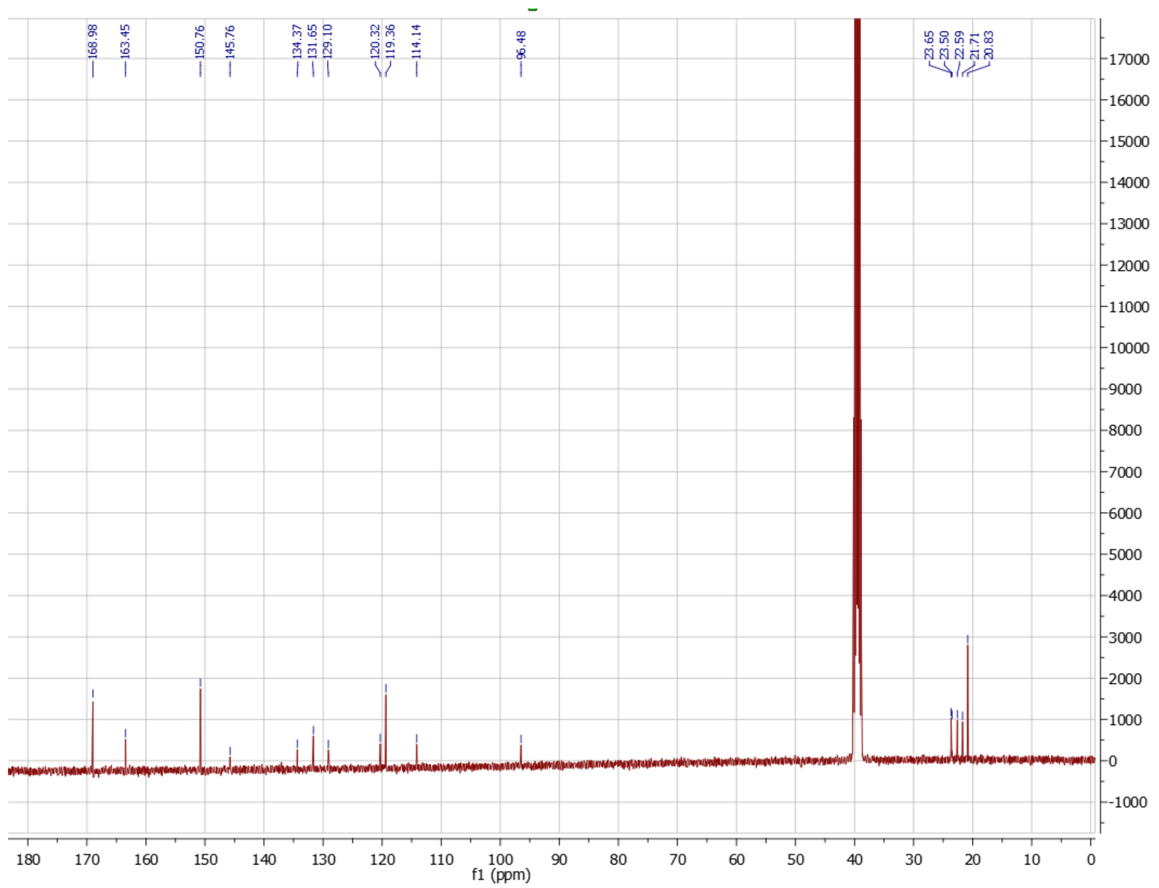

Figure S5. ^{13}C NMR spectrum for compound 3.

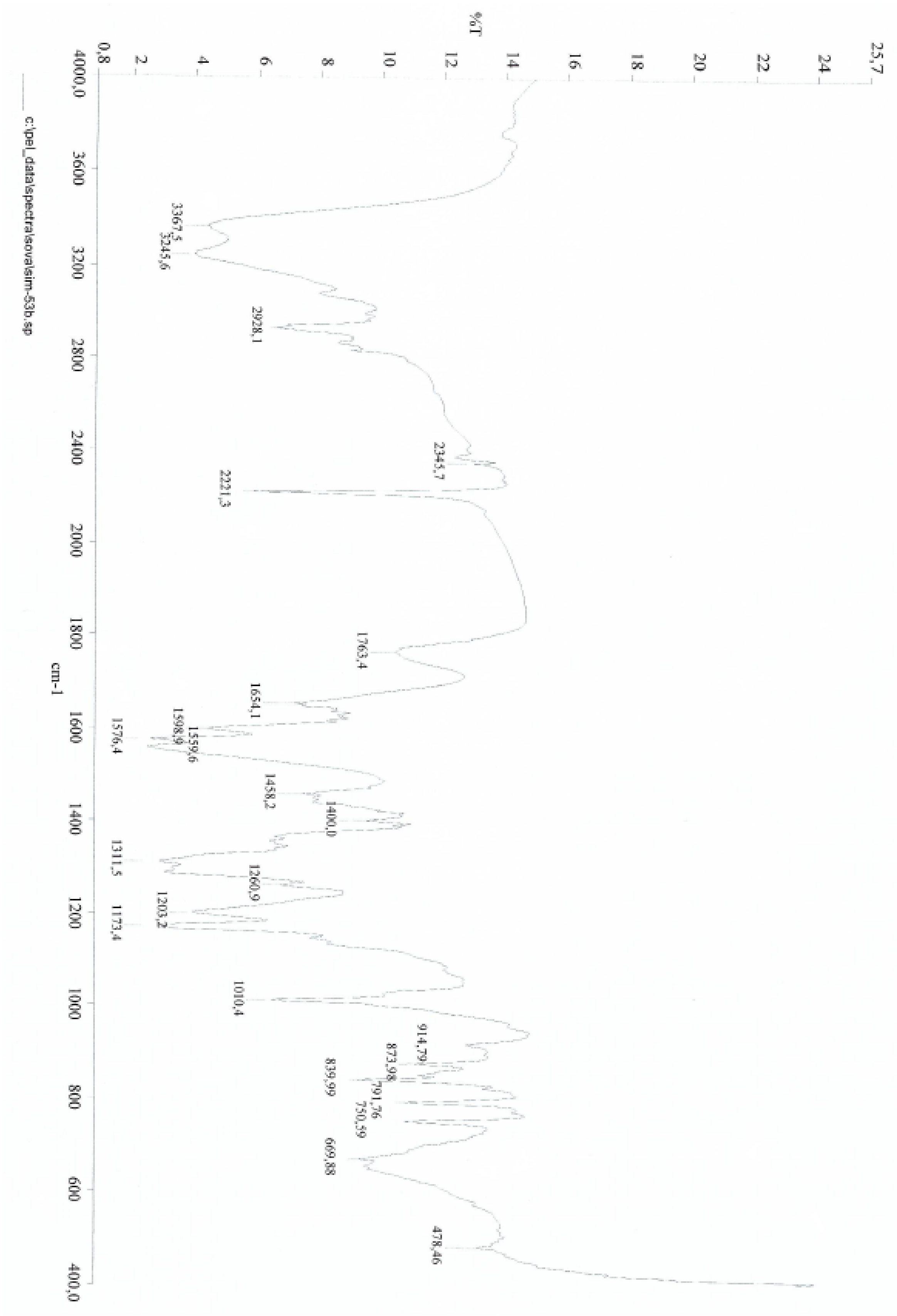

Figure S6. IR spectrum for SIM-53B.

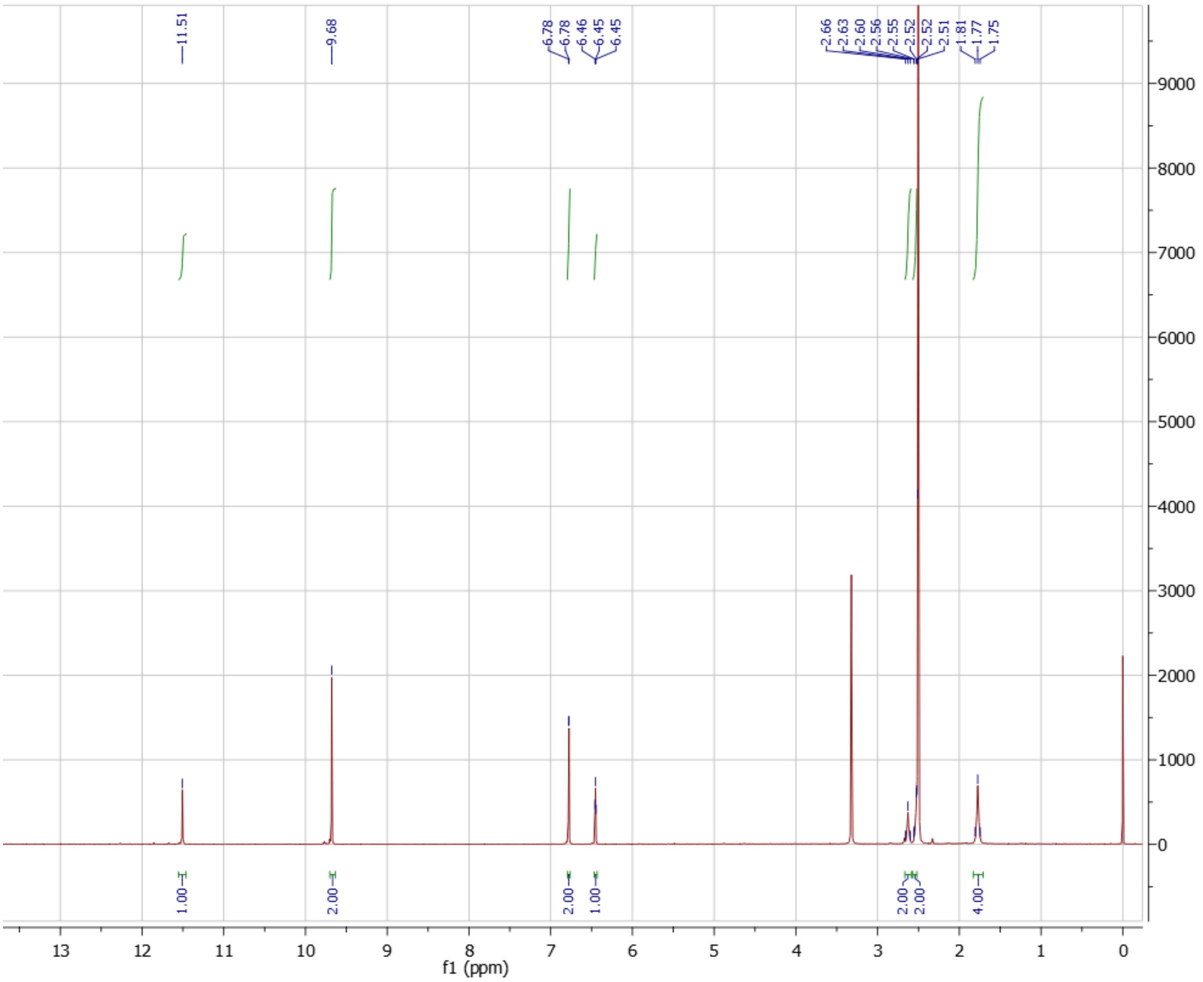

Figure S7. ^{1}H NMR spectrum for SIM-53B.

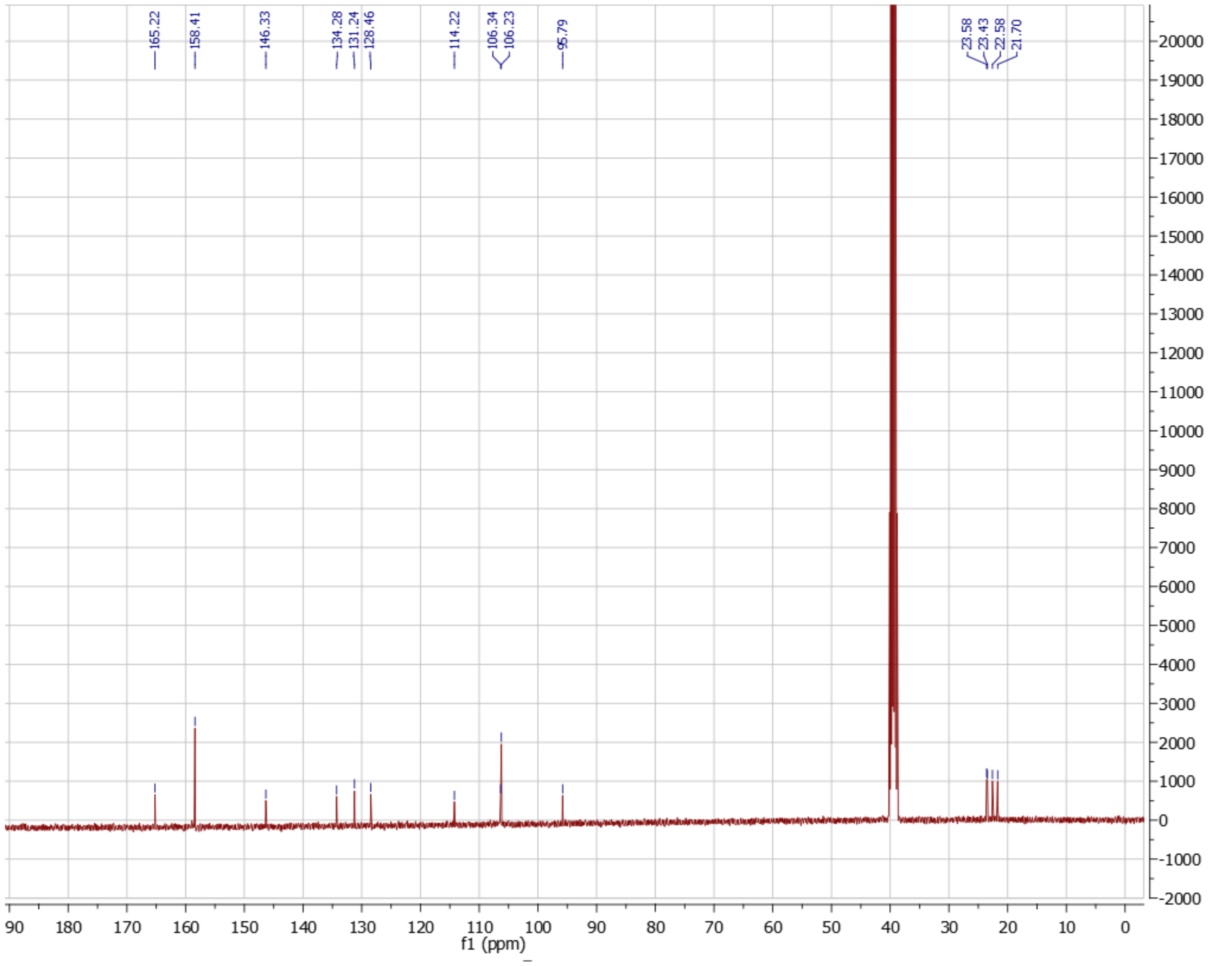

Figure S8. ^{13}C NMR spectrum for SIM-53B.

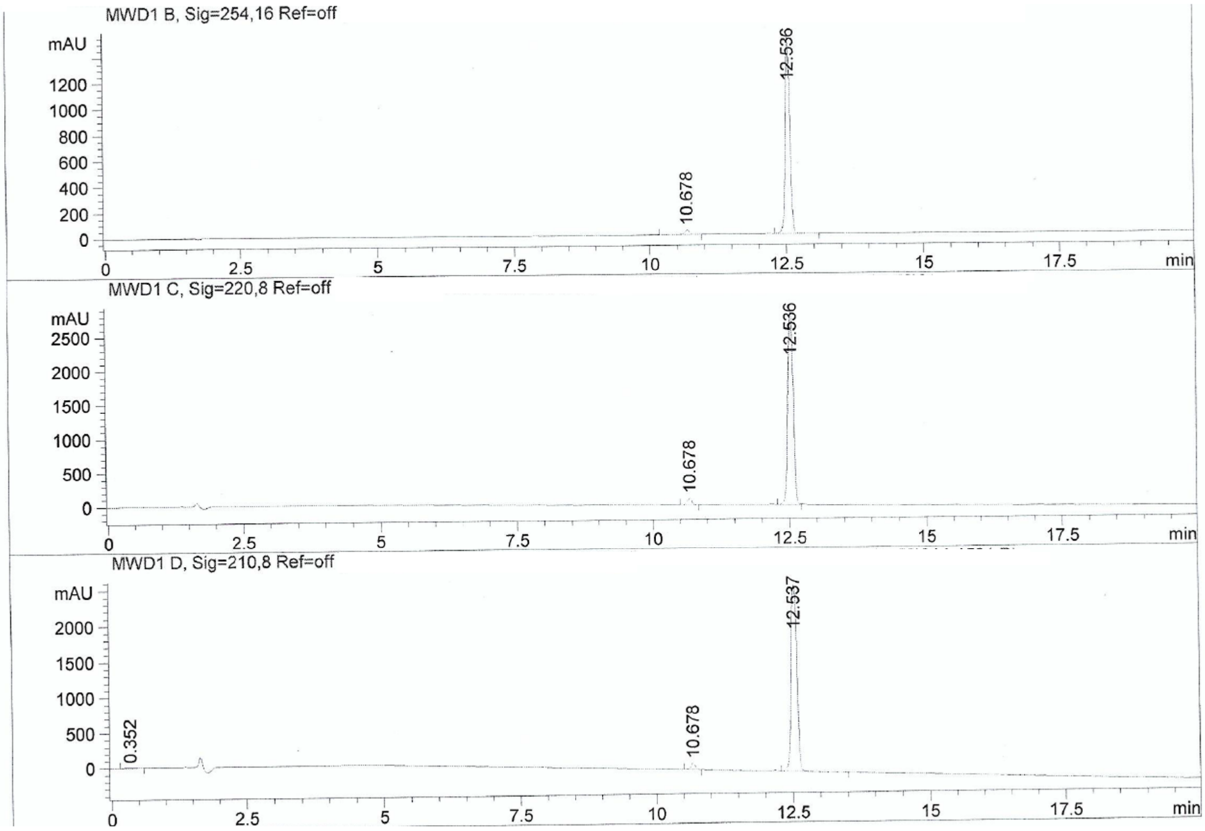
MWD1 B, Sig=254,16 Ref=off
mAU
1200
1000
800
600
400
200
0
10.678
12.536
0
2.5
5
7.5
10
12.5
15
17.5
min
MWD1 C, Sig=220,8 Ref=off
mAU
2500
2000
1500
1000
500
0
10.678
12.536
0
2.5
5
7.5
10
12.5
15
17.5
min
MWD1 D, Sig=210,8 Ref=off
mAU
2000
1500
1000
500
0
0.352
10.678
12.537
0
2.5
5
7.5
10
12.5
15
17.5
min

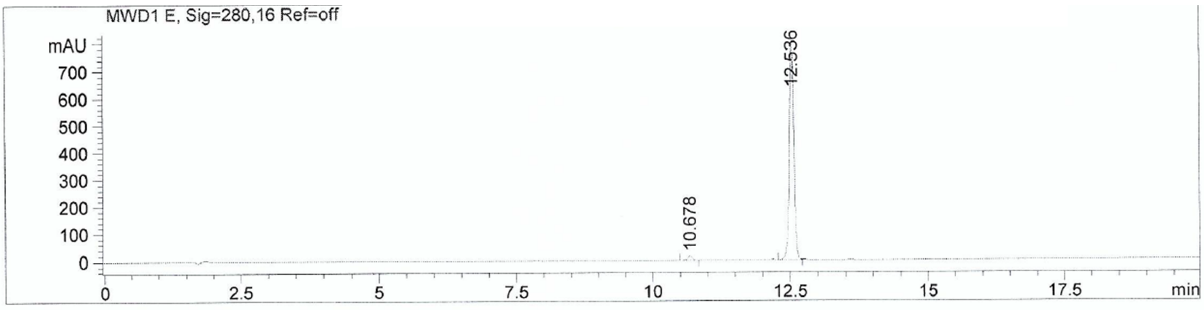
MWD1 E, Sig=280,16 Ref=off
mAU
700
600
500
400
300
200
100
0
10.678
12.536
0
2.5
5
7.5
10
12.5
15
17.5
min

Area Percent Report

Sorted By : Signal
Multiplier : 1.0000
Dilution : 1.0000
Use Multiplier & Dilution Factor with ISTDs

Signal 1: MWD1 B, Sig=254,16 Ref=off

Peak #	RetTime [min]	Type	Width [min]	Area [mAU*s]	Height [mAU]	Area %
1	10.678	BV	0.0933	212.07198	34.69529	2.3804
2	12.536	VB	0.0887	8696.84473	1520.97827	97.6196
Totals :				8908.91670	1555.67356	

Signal 2: MWD1 C, Sig=220,8 Ref=off

Peak #	RetTime [min]	Type	Width [min]	Area [mAU*s]	Height [mAU]	Area %
1	10.678	VB	0.0915	555.66302	93.26781	2.8134
2	12.536	VV	0.1086	1.91949e4	2847.49634	97.1866
Totals :				1.97505e4	2940.76414	

Signal 3: MWD1 D, Sig=210,8 Ref=off

Peak #	RetTime [min]	Type	Width [min]	Area [mAU*s]	Height [mAU]	Area %
1	0.352	BB	0.2437	59.15961	3.52653	0.2940
2	10.678	BB	0.0906	546.41016	92.88100	2.7150
3	12.537	VV	0.1203	1.95201e4	2638.31055	96.9911
Totals :				2.01256e4	2734.71807	

Signal 4: MWD1 E, Sig=280,16 Ref=off

Peak #	RetTime [min]	Type	Width [min]	Area [mAU*s]	Height [mAU]	Area %
1	10.678	VB	0.0923	78.34251	12.99612	1.6955
2	12.536	VV	0.0886	4542.14209	795.47021	98.3045
Totals :				4620.48460	808.46634	

*** End of Report ***

Figure S9. HPLC chromatogram and area percent report for SIM-53B.

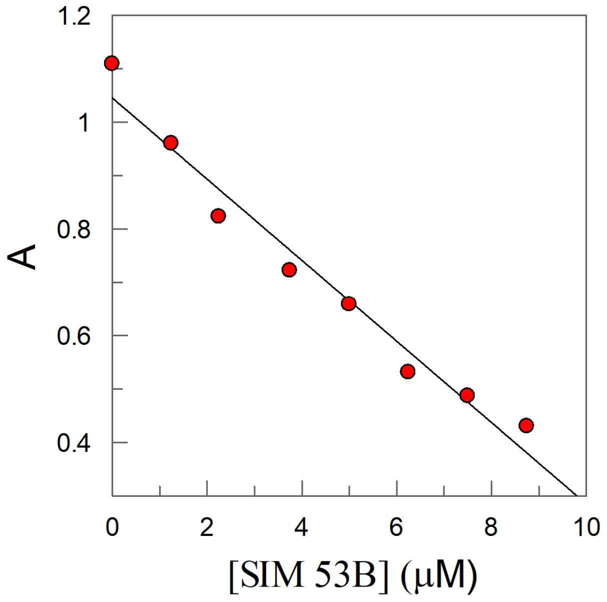

Figure S10. Antioxidant activity determined by ABTS: calibration curve. Changes of the absorbance of initial $ABTS^{•+}$ radical remaining after the reaction with seven different concentrations of SIM-53B (1.25 – 8.75 μM of SIM-53B) at time 90 min and room temperature.

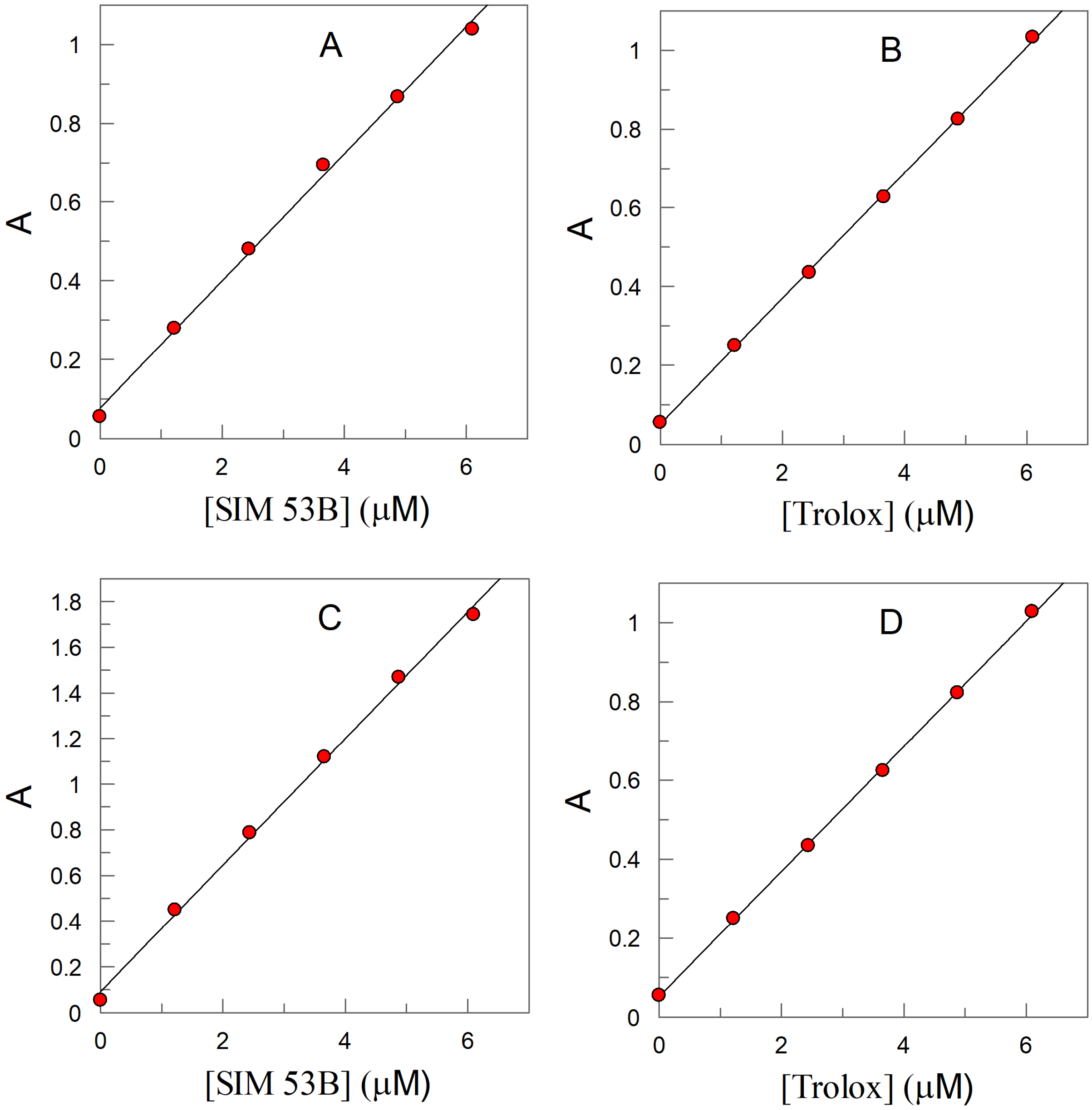

Figure S11. CUPRAC assay: calibration curves. Calibration curves employed to determine $TEAC_{CUPRAC}$ values at room temperature (A and B) and at T = 50 ºC (C and D).
